# Some Human Lessons in War-Time

**Published:** 1916-09-30

**Authors:** 


### LIFE AND DEATH.

### Some Human Lessons in War Time.

### II. THE PHYSICIAN AND THE PREACHER.*

How many men and women are there who have devoted continuous thought and a life-study to the causes which make for and against mental death in their own lives and in the daily life of the individual through all classes of the people? All that tends to defeat spiritual death and rescue man or woman from it should result in increased vitality for each individual who has mastered the underlying causes which make for spiritual life and health and eternity. A study of national history and of the social life of the people in these islands cannot fail to impress the knowledgeable student with the conviction that our forbears and sires had a keener sense of spirituality than the majority of us have to-day. While extraordinary additions have been made, especially during the last fifty or sixty years, to our wealth, our knowledge, and our command over the forces of Nature, we confess to the feeling that our spiritual faculties have become greatly impaired, and that this generation has not the vision of those who lived in simpler times.

Where is the preacher to-day who insistently, or even at all, teaches that unless we are wholesome in spirit we cannot be healthy in body? This truth was recognised and enforced by the preachers and leaders of a former generation and also by their predecessors. Our experience of life has brought home to us the truth, which is continuously enforced by our intercourse with our fellows, that the most important of all things to men and women from the day of adolescence onwards is a keen apprehension of their spirituality. The spiritual side of man or woman has a most vital effect upon the well-being of each. Those who have the Spirit have life; those who have never been aroused to the existence of their spirituality as a real living power and source of strength and influence to men and women, tend to be more and more animal in the sense that their thoughts, their interests, their actions, their desires, their ambitions, their aims and outlook become restricted and confined to those things which, in their view, tend to bring them the maximum of earthly prosperity and to give them what is colloquially described as "a good time."

In practice, as every medical practitioner knows, the man without spirituality, that is without the apprehension of the existence of the spiritual side of life, is not pursuing a course of existence which will tend to promote the maximum of vitality and the continuous development, purification, and strength of his physical being. Given a man or woman who believes in immortality, who realises that physical life on earth is a dying life from its commencement to its termination, and who prays to God with an assured conviction of His great mercy through Christ, and we have a personality which demonstrates in a myriad of ways that to be wholesome in spirit is to be healthy in body. Further, such a personality impresses all who have the good fortune to meet it with courage, forcefulness, transparent honesty, with the purposeful, energetic, and continuous pursuit of truth, for truth's sake, the never-ceasing desire to utilise every available opportunity without pretension, or self-assertion, or anything but the most considerate regard for the feelings of others, to labour continuously to make the lot of every person with whom it comes in contact happier, and to leave the world a little better than each has found it. Individuals with such a personality have the vision which many of this generation before the war had not.

There are many good and earnest men amongst the clergy and ministers of religion in this country who are undoubtedly wholesome in spirit, but are there not many others who, when tested by the atmosphere and conduct of the church services for which they are responsible, and the character and subjects of their sermons at the outbreak of war, could not be recognised as being so? A few years ago the press was occupied with a religious census, the purpose of which was, we believe, to show that places of worship were less frequented than was the case at an earlier date. To-day, too, there is a general complaint amongst religious teachers that the attendance at churches and places of worship on Sunday, as on week-days, is relatively small. If these clerical complainants were fully alive and awake to their own spirituality they would not spend words or waste time on idle regrets. On the contrary, with the personality of the true believer, and as a man charged with vigorous life and energy through his belief in Christ, each teacher would go straight to the root of the matter. Then, with an awakened sense of his spiritual nature he might reject as unworthy the habit of mere existence which had choked his energies and blinded him to his responsibility to his people. He might become a living force full of the Holy Spirit, having the old vision of Eternal Truth, and so make his church once more what it never ought to have ceased to be—the Church of the Living God. He might shed the tepid philosophy of the German unbeliever whom many preachers in recent years have quoted in their pulpits as evidence of their wide reading and surpassing knowledge—save the mark! He might turn his own thoughts and direct also those of his people to the truths of the Living God, to the teachings and life of Christ, and to the overwhelming call there is to the people of the British nation to unite in intercession to Almighty God that the outcome of the present war may be the awakening to Spiritual Life of all classes of the people and the extension of Christ's kingdom on earth.

* The previous article appeared on September 23.

How many of the younger clergy, like our warriors in both Services who have fought so bravely and nobly in our defence, have had the vision and responded to its call by giving up their comfortable home charges to go out to the Front to help supply the spiritual needs of our fighting men? In all the story of the war two types of men are most striking and wonderful. Both of them have been characterised by an entire forgetfulness of self, a total disregard of the dangers and risks of the fighting line, an utter surrender of self, and an assured conviction, through their spiritual nature, that the steady pursuance of their daily duty in the face of the enemy held them safe, though surrounded by horrors and risks greater than any men probably have ever had to face before in the history of the world, because of the immortal truth that the place of duty is the place of safety. The two types of men are our warriors, and the minister of God, to whatever denomination he may belong, who is known at the Front as the "padre," for there the representatives of all creeds unite and co-operate in the most perfect amity while risking their lives and offering the whole of their energies and of themselves to the cause of Christ and His work for men.

Would that the same spirit might come to-day to the "padre" in every pulpit throughout the Homeland, for then the churches would not contain seating accommodation enough for the congregations which would speedily fill them at every service, and the people still left at home in the Old Country would assuredly, like the "padres" of all denominations and our fighting men, become conscious of their spiritual nature.

Then everywhere throughout the nation the atmosphere would be charged with the living forces without which the home-coming of our warriors after the war may prove to them startlingly cold, because we home residents, with our spiritual leaders, have failed to remember that Almighty God is present always in His house, and that it is the privilege, and the proof of the vitality of each man and woman, habitually to attend there to express thanksgiving for the vision which has come to them and our warriors, and to renew their strength. The war has reduced enormously the number of patients of many practitioners, whose consulting-rooms were formerly crowded. One cause of this is the sincere awakening of an ever-increasing number of all classes of the population to the feeling that they must arouse themselves and devote time and energy to war work. Our women, like our men, have shown great self-denial and a noble spirit since they have become seized with a consciousness of the issues which underlay the outbreak of this terrible war. Is it not possible to bring the vision home to every clergyman and minister of religion throughout the land, that he may devote his whole strength, with God's help, to realise the vision for himself and to bring it home to the souls of the people of his communion? Why do so many fail to arouse in their congregations the keenest sense of their spiritual nature, and to recognise that unless every one of us is wholesome in spirit he cannot be healthy in body?

Some there may be who refuse to recognise the close connection which has prevailed for centuries between the priest and minister of religion and the practitioner of medicine. They may hold grave doubts as to the connection between spirituality and a healthy body; they may have missed their way, or become surfeited with convention and routine, so that to them the very idea of a vigorous life is made hateful. Ought they not to accept such feelings as evidence that they may have approached so near to the death of their spiritual nature that it has become almost impossible for them to put on the Spirit? We will ask them, if any such patients there be, to face this question: Why will ye die spiritually? All around you, in increasing volume, there must be evidence of the renewal of the Spirit in growing numbers of the population, and in people of all ages and classes and types with whom you mingle. Surely the boasted intelligence of an unbeliever must be startled and aroused by the stir and product of the forces at work in their neighbours and amongst their friends. Make the effort. Cry a halt. Shed mere existence. Take on vitality. Seek God's help, cultivate the wholesome spirit, and a life's experience emboldens us to say you may, if you will, become healthy in body, fervent in spirit and blessed in all things.

**Next week's article will deal with "Childhood and the Children."**

